# Supplementary material for: SCALE method for single-cell ATAC-seq analysis via latent feature extraction
Source: Nat Commun. 2019 Oct 8;10:4576. doi: 10.1038/s41467-019-12630-7 (PMC6783552; doi:10.1038/s41467-019-12630-7)
Supplement: Supplementary file 1 — Supplementary Information [file 41467_2019_12630_MOESM1_ESM.pdf]

Supplementary Information for

Single-cell ATAC-seq analysis via latent feature extraction

Lei Xiong, Kui Xu, Kang Tian, Yanqiu Shao, Lei Tang, Ge Gao, Michael Zhang, Tao Jiang, Qiangfeng Cliff Zhang

Correspondence to: Q.C.Z. (qczhang@tsinghua.edu.cn)

This PDF file includes:

Supplementary Figure 1 | scATAC-seq datasets used in this study.
Supplementary Figure 2 | Feature embedding.
Supplementary Figure 3 | Visualization of trajectory relationships with UMAP.
Supplementary Figure 4 | Clustering accuracy by confusion matrix.
Supplementary Figure 5 | Analysis of clustering results.
Supplementary Figure 6 | Impact of data corruption on clustering accuracy on six datasets.
Supplementary Figure 7 | Results of different cluster number (k) estimated by SCALE.
Supplementary Figure 8 | Imputation efficiency on the six real datasets.
Supplementary Figure 9 | Inter and intra-correlation of subgroups of the raw and the imputed data.
Supplementary Figure 10 | Cluster-specific peaks of raw, imputed and binary imputed data.
Supplementary Figure 11 | Imputation improves the identification of cell type-specific motifs with chromVAR.
Supplementary Figure 12 | Imputation results on the six real datasets at different corruption levels.
Supplementary Figure 13 | Data corruption impact on preserving original data structure.
Supplementary Figure 14 | Imputation results on the simulation dataset at different corruption levels.
Supplementary Figure 15 | Embedding and specific peaks of the CD45+ and the Epcam+ cells.
Supplementary Figure 16 | Feature-associated peaks.
Supplementary Figure 17 | PCA/SCALE extracted features of the GM12878 cells from the InSilico dataset.
Supplementary Figure 18 | SCALE extracted features of the Splenocyte dataset.
Supplementary Figure 19 | SCALE extracted features of the Forebrain dataset.
Supplementary Figure 20 | Results of SCALE on a mouse atlas dataset.
Supplementary Figure 21 | Running time and memory.

Supplementary Table 1 | Results of SCALE with different encoder structures and latent dimensions.
Supplementary Table 2 | Statistics of scATAC-seq datasets.

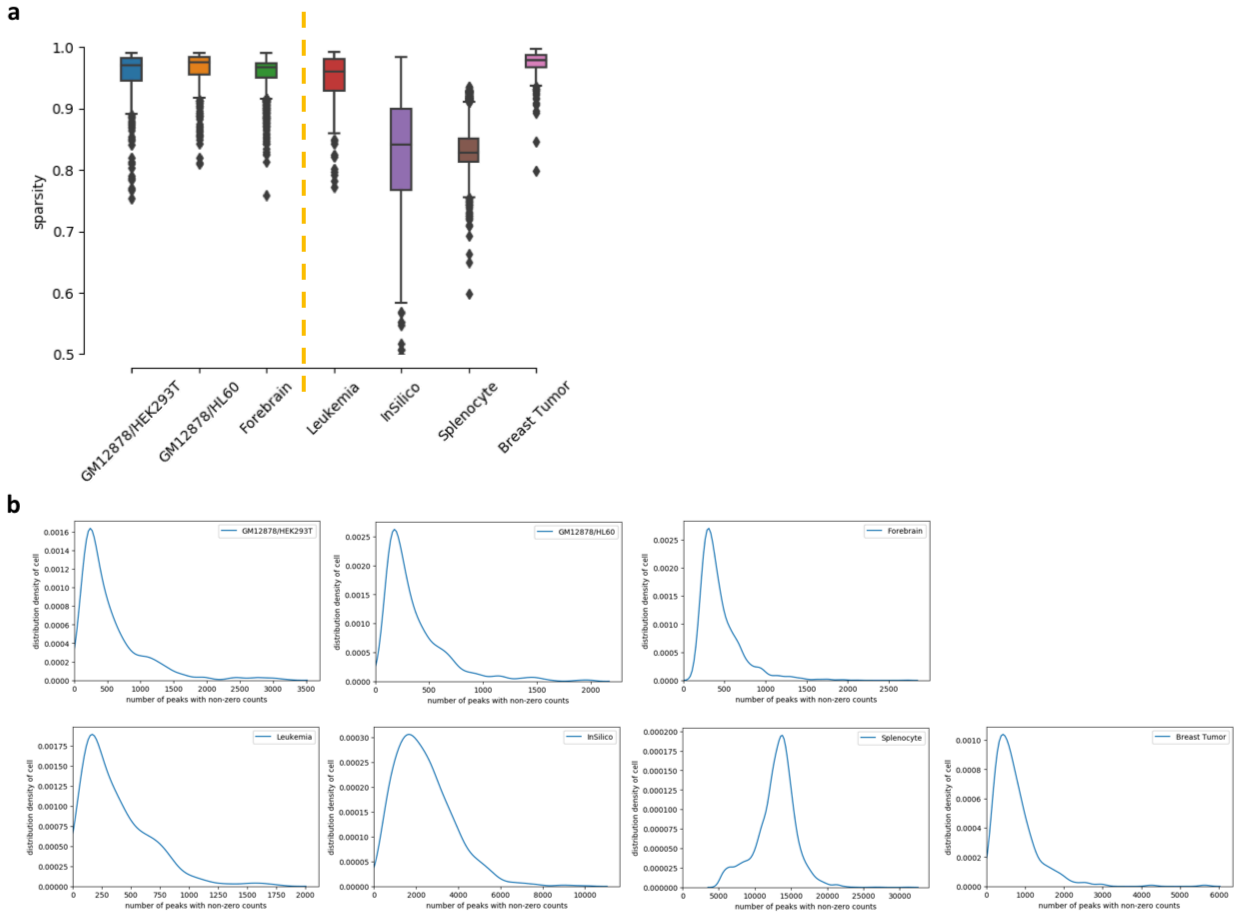

Supplementary Figure 1 | scATAC-seq datasets used in this study. a, Boxplot of data sparsity of seven datasets (GM12878/HEK293T, GM12878/HL-60, Forebrain, Leukemia, InSilico, Splenocyte, and Breast Tumor. Datasets are separated with yellow dashed lines by distinct scATAC-seq platforms). We used the proportion of zero-valued peaks to define the sparsity for each cell. **b,** Distribution of the number of cells with non-zero peaks for the seven datasets.

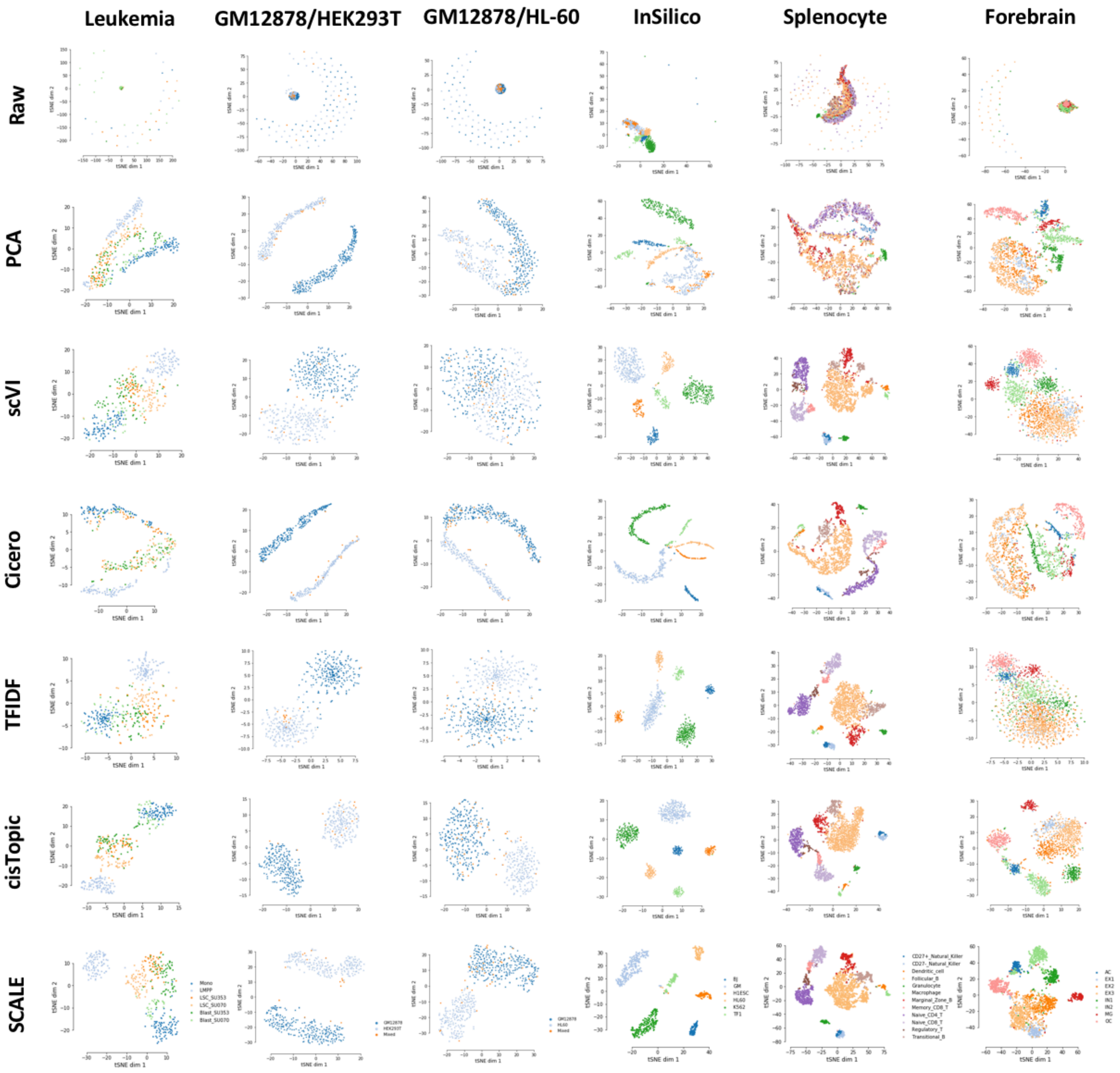

Supplementary Figure 2 | Feature embedding. *t*-SNE visualization of the raw data and the extracted features from PCA, scVI, Cicero, TF-IDF, cisTopic and SCALE on the training Leukemia dataset and the testing datasets including GM12878/HEK293T, GM12878/HL-60, InSilico, Splenocyte, and Forebrain. For comparison, SCALE, PCA and scVI all performed dimension reduction to ten dimensions before applying *t*-SNE while the raw data were directly visualized with *t*-SNE.

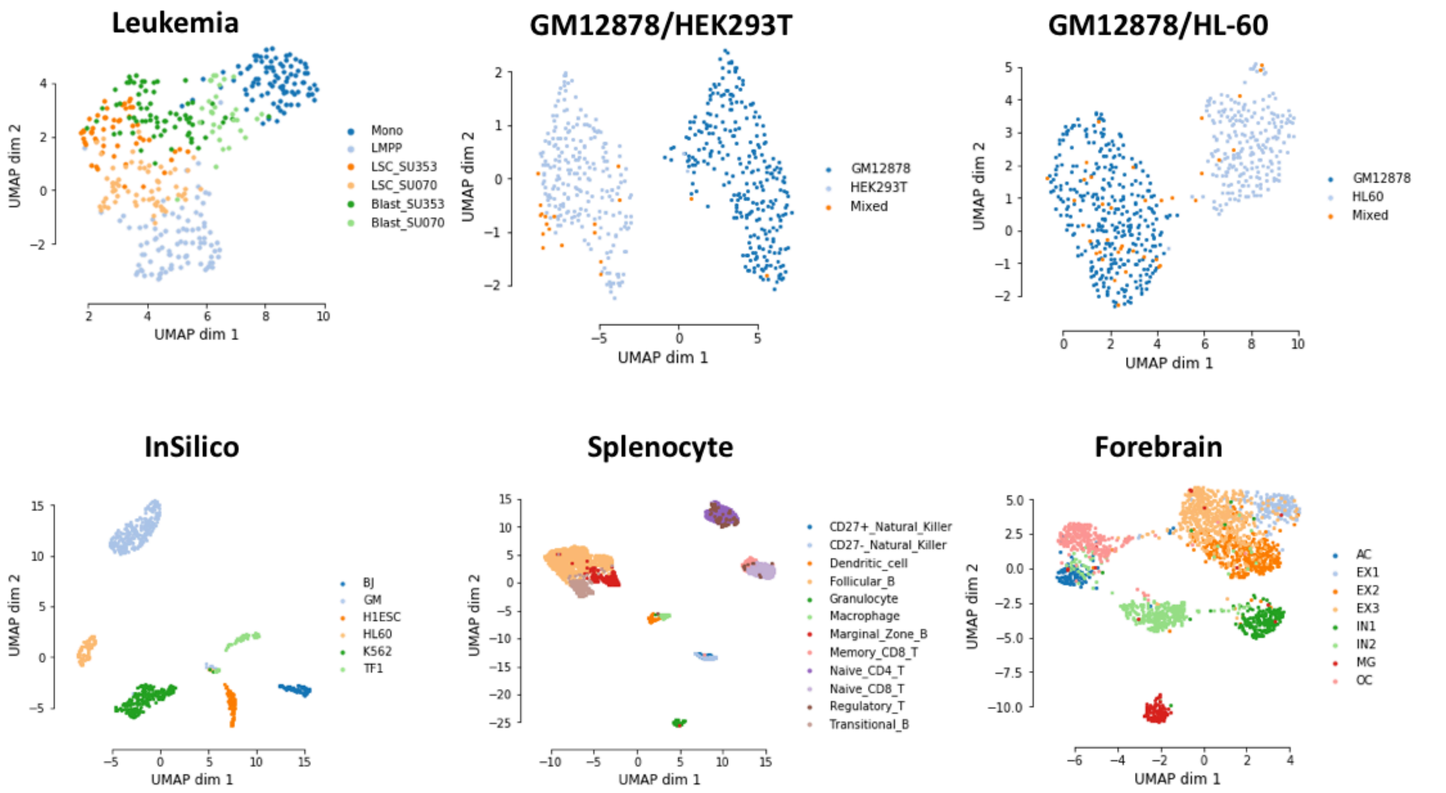

Supplementary Figure 3 | Visualization of trajectory relationships with UMAP. UMAP visualization of the extracted features from SCALE on the Leukemia, GM12878/HEK293T, GM12878/HL-60, InSilico, Splenocyte, and Forebrain datasets.

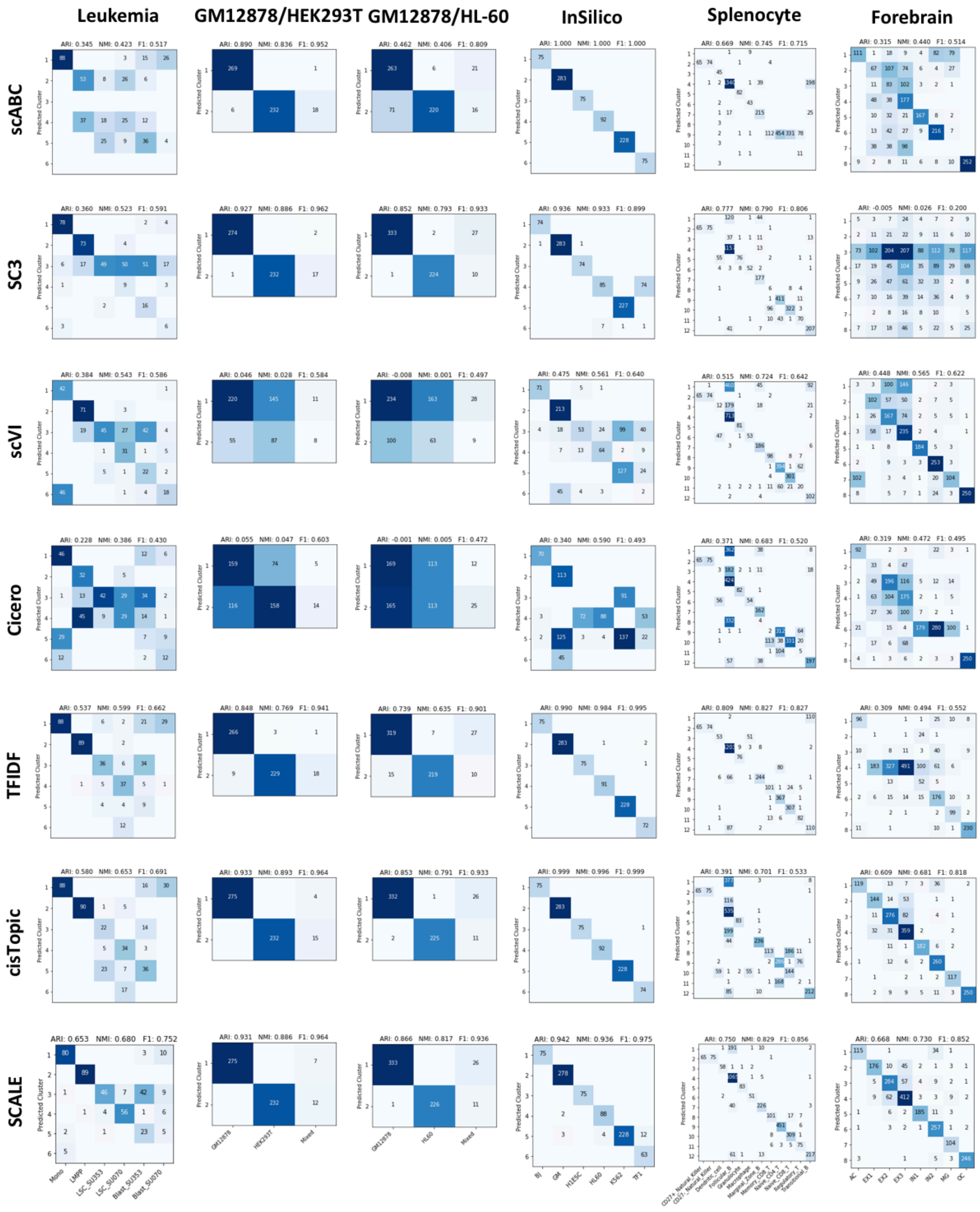

Supplementary Figure 4 | Clustering accuracy by confusion matrix. Clustering accuracy was evaluated by confusion matrices between reference cell types and cluster assignments predicted by scABC, SC3, scVI, Cicero, TF-IDF, cisTopic and SCALE. For scABC and SC3 the cluster assignments were directly obtained from the output of the tools; for SCALE and scVI, we applied the *K*-means clustering on the extracted ten features to obtain cluster assignments. ARI, NMI and F1 scores are shown on the top.

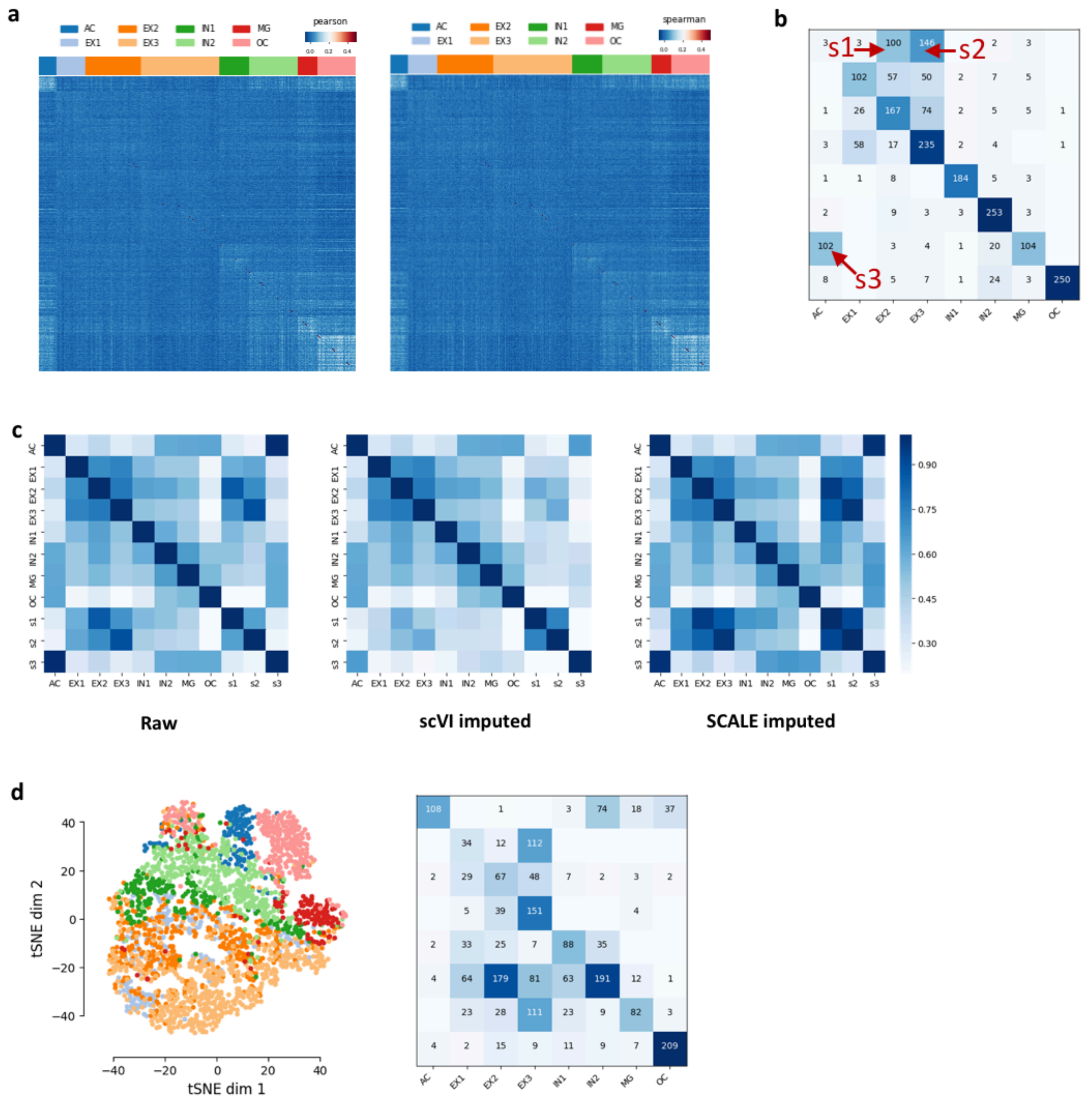

Supplementary Figure 5 | Analysis of clustering results. a, Ill-defined cell-wise Pearson and Spearman distance matrices of the Forebrain dataset. **b,** The confusion matrix between the scVI clustering results and the reference cell types. The three subgroup *s1*, *s2* and *s3* deviated the most from reference labels. **c,** Pearson correlation coefficients of the meta cells of the eight cell types of the Forebrain dataset plus three cell subgroups (*s1*, *s2*, *s3*) in the raw dataset, the scVI imputed data, and the SCALE imputed data, respectively. **d,** The feature embedding and the confusion matrix of clustering results of the Forebrain dataset by SCALE without GMM.

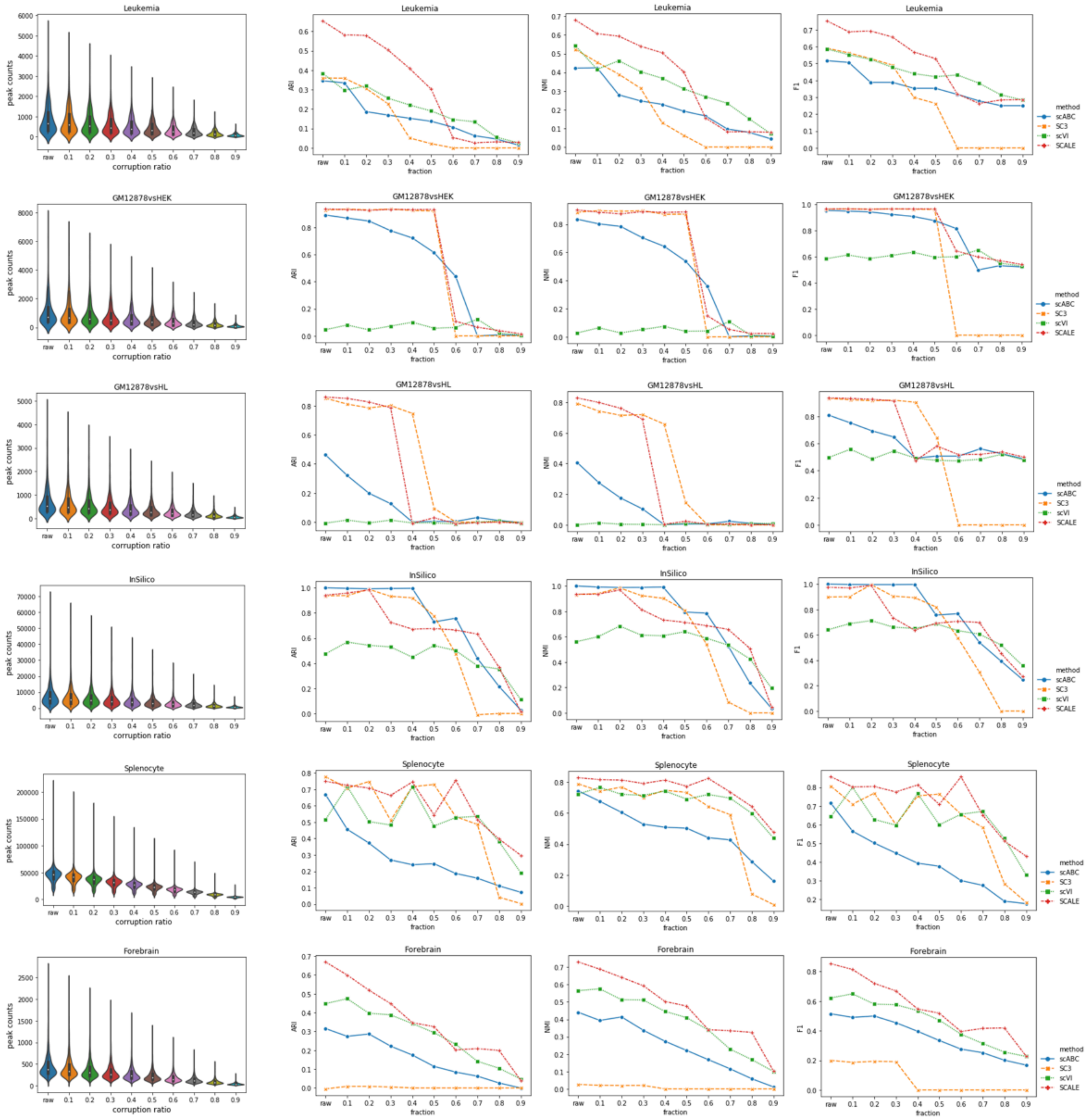

Supplementary Figure 6 | Impact of data corruption on clustering accuracy on six datasets. Corrupted data at a different ratio (0.1-0.9, left panel) and bar plots of the Adjusted Rand Index (ARI), the Normalized Mutual Information (NMI) and the F1 score to measure clustering accuracy of different clustering methods respectively.

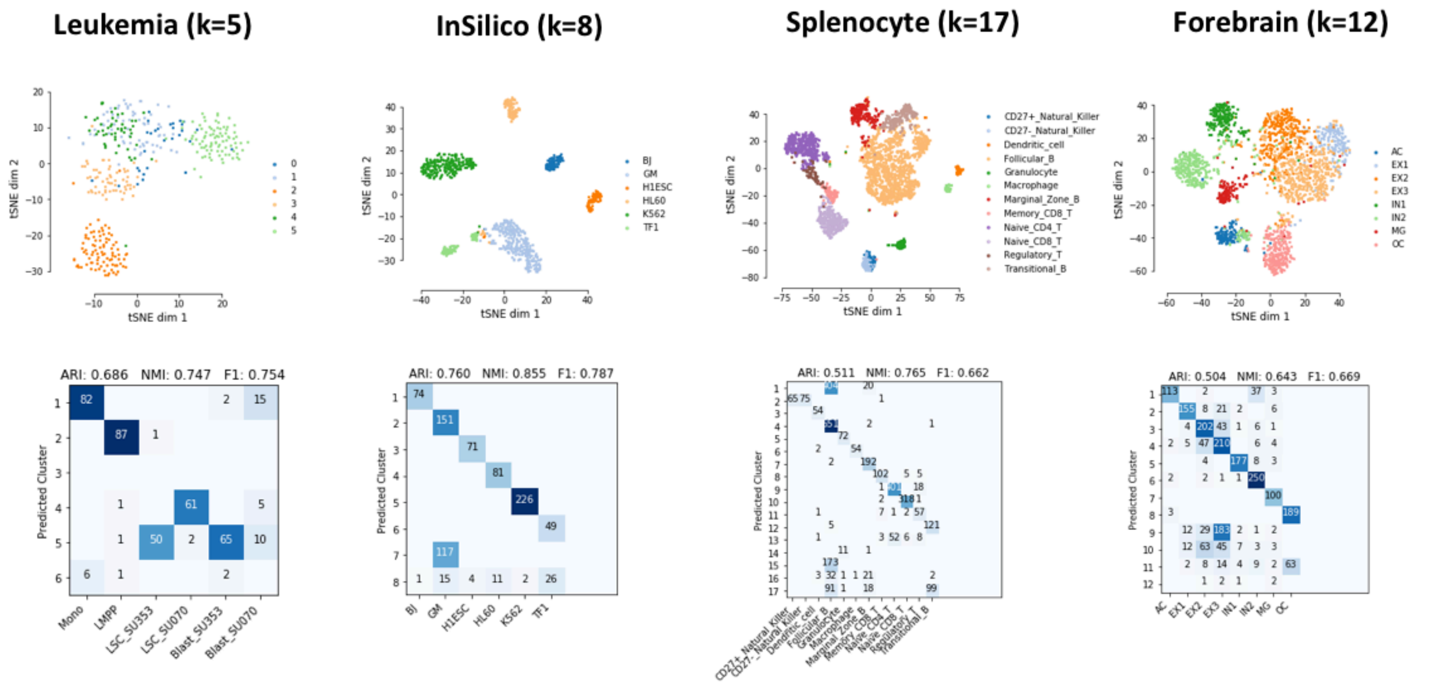

Supplementary Figure 7 | Results of different cluster number (k) estimated by SCALE. *t*-SNE visualization and cluster confusion matrix of Leukemia (k=5), InSilico (k=8), Splenocyte (k=17) and Forebrain (k=12).

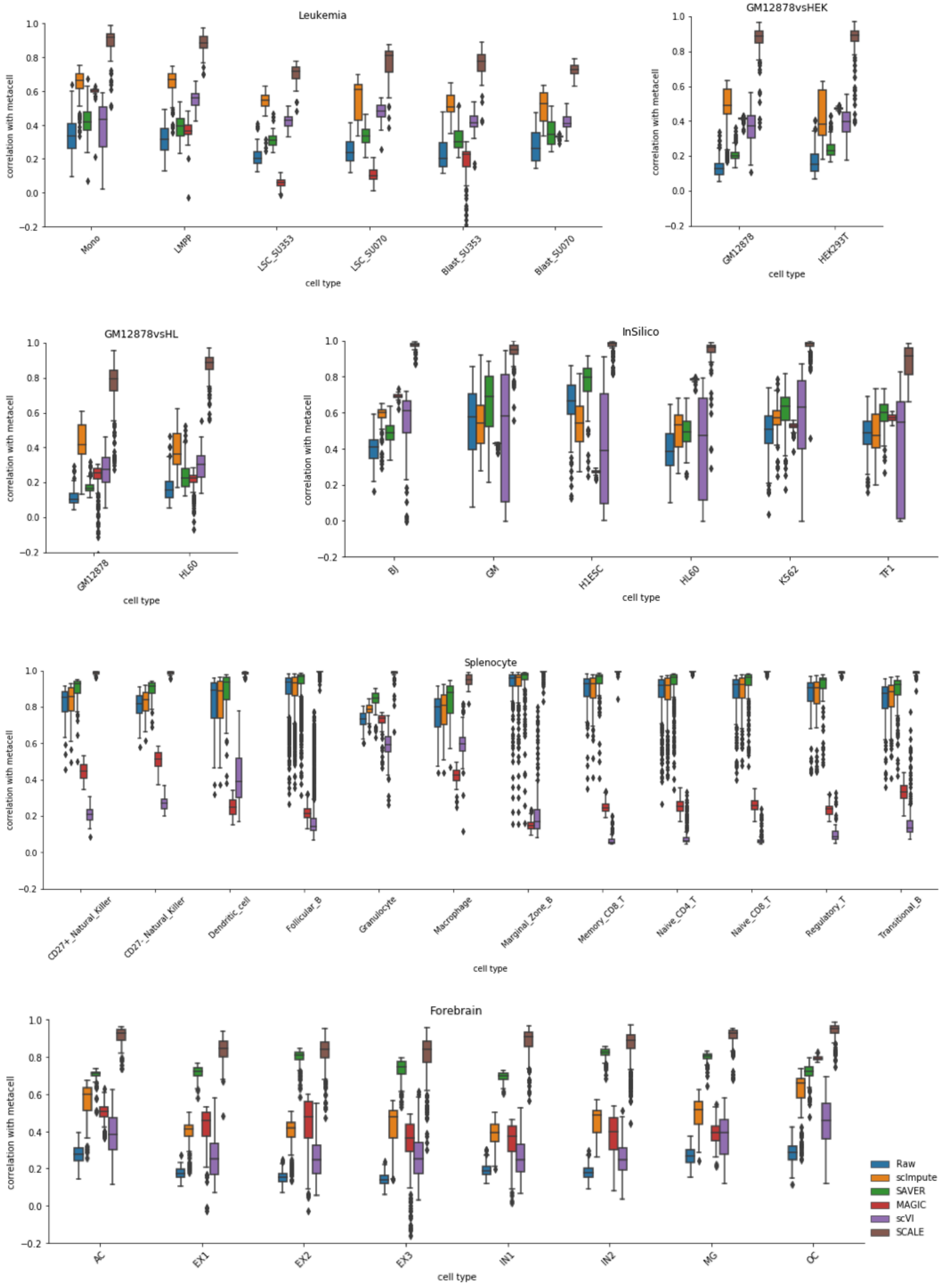

Supplementary Figure 8 | Imputation efficiency on the six real datasets. Cell-wise correlations of the imputed data with the meta cell of each cell type for comparison.

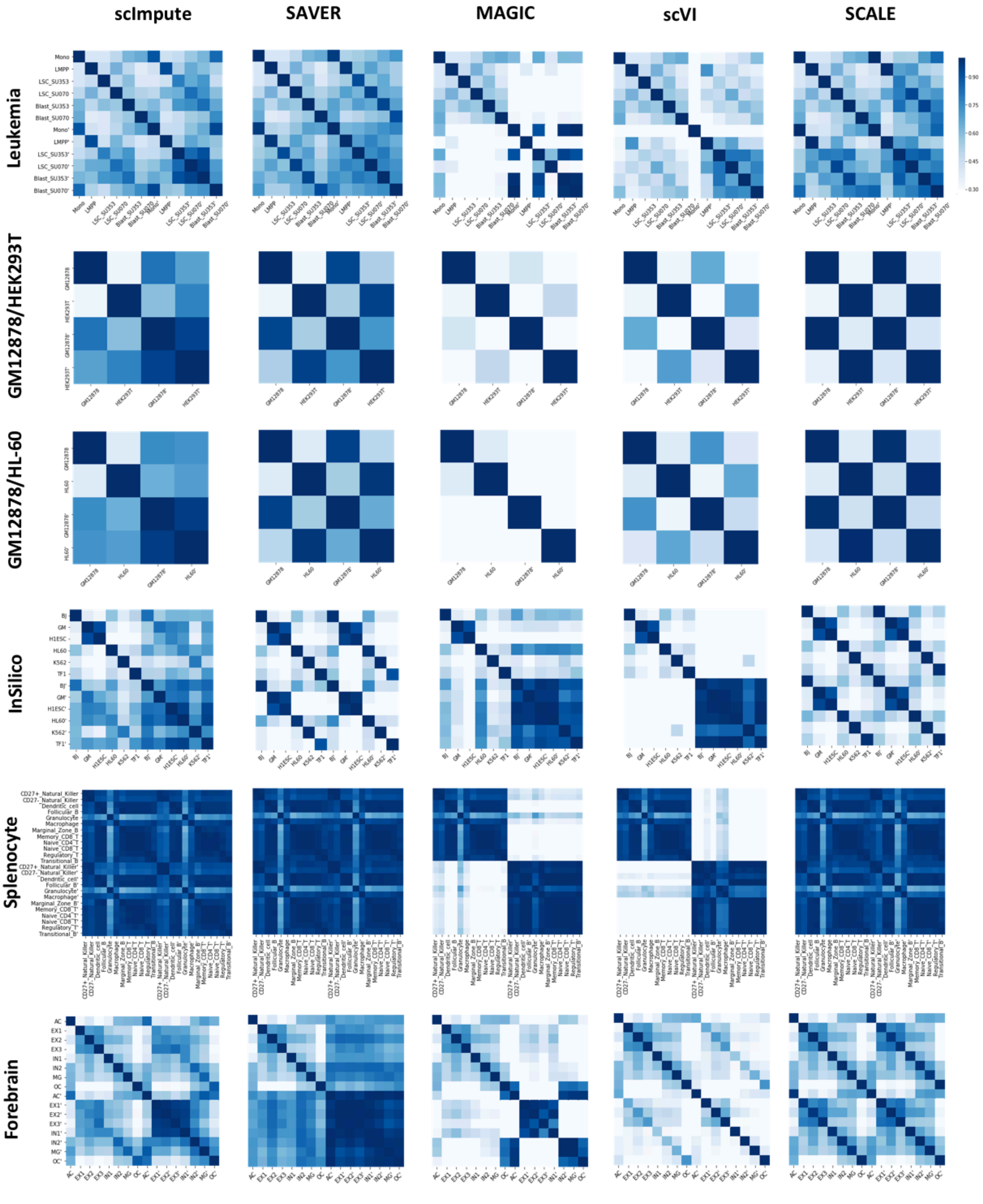

Supplementary Figure 9 | Inter and intra-correlation of subgroups of the raw and the imputed data. Pearson correlation coefficients among the meta cells of the different cell types of the raw data and the different clusters in the imputed data.

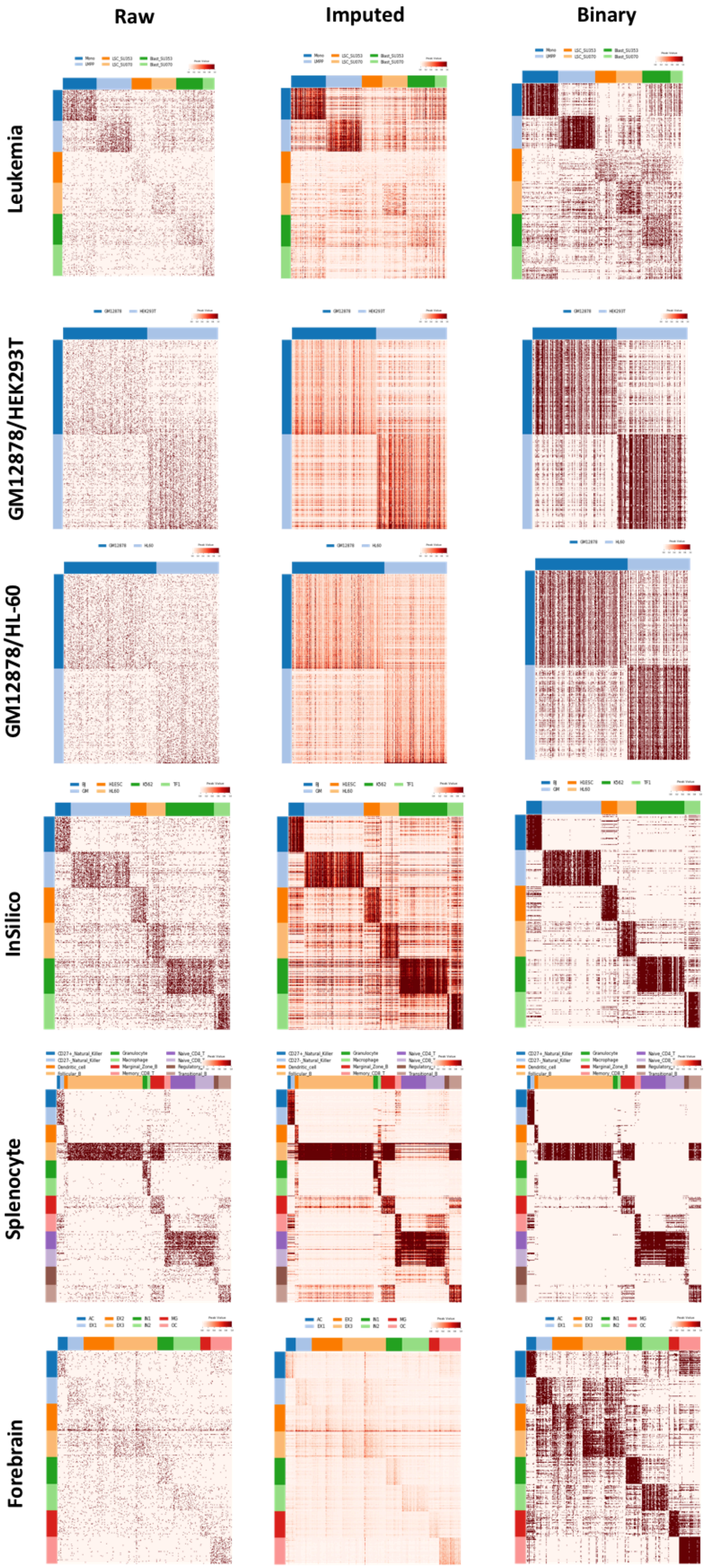

Supplementary Figure 10 | Cluster-specific peaks of raw, imputed and binary imputed data. Top 200 specific peaks for each cluster, and compared imputed and binary imputed data on these peaks.

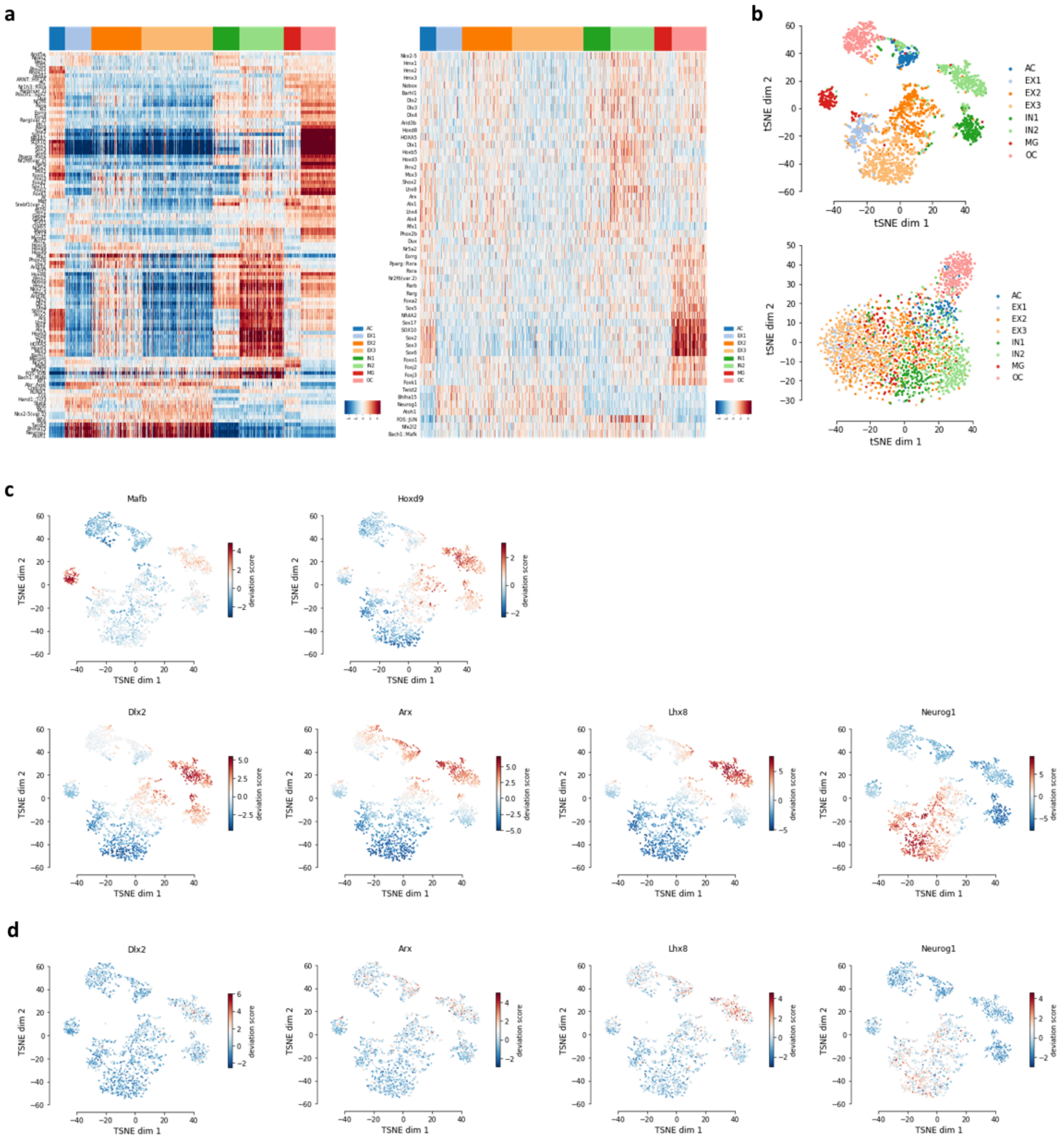

Supplementary Figure 11 | Imputation improves the identification of cell type-specific motifs with chromVAR. a, 105 significant motifs profile of imputed data and 52 motifs profile of raw data identified by chromVAR on 4100 differential accessible peaks of Forebrain data. **b,** *t*-SNE embeddings of motifs profile of imputed and raw data. **c, d,** embeddings of motifs only identified by imputed data Mafb, Hoxd9 and motifs identified by both imputed and raw data Dlx2, Arx, Lhxh8 and Neurog1 colored by deviation scores of chromVAR**.**

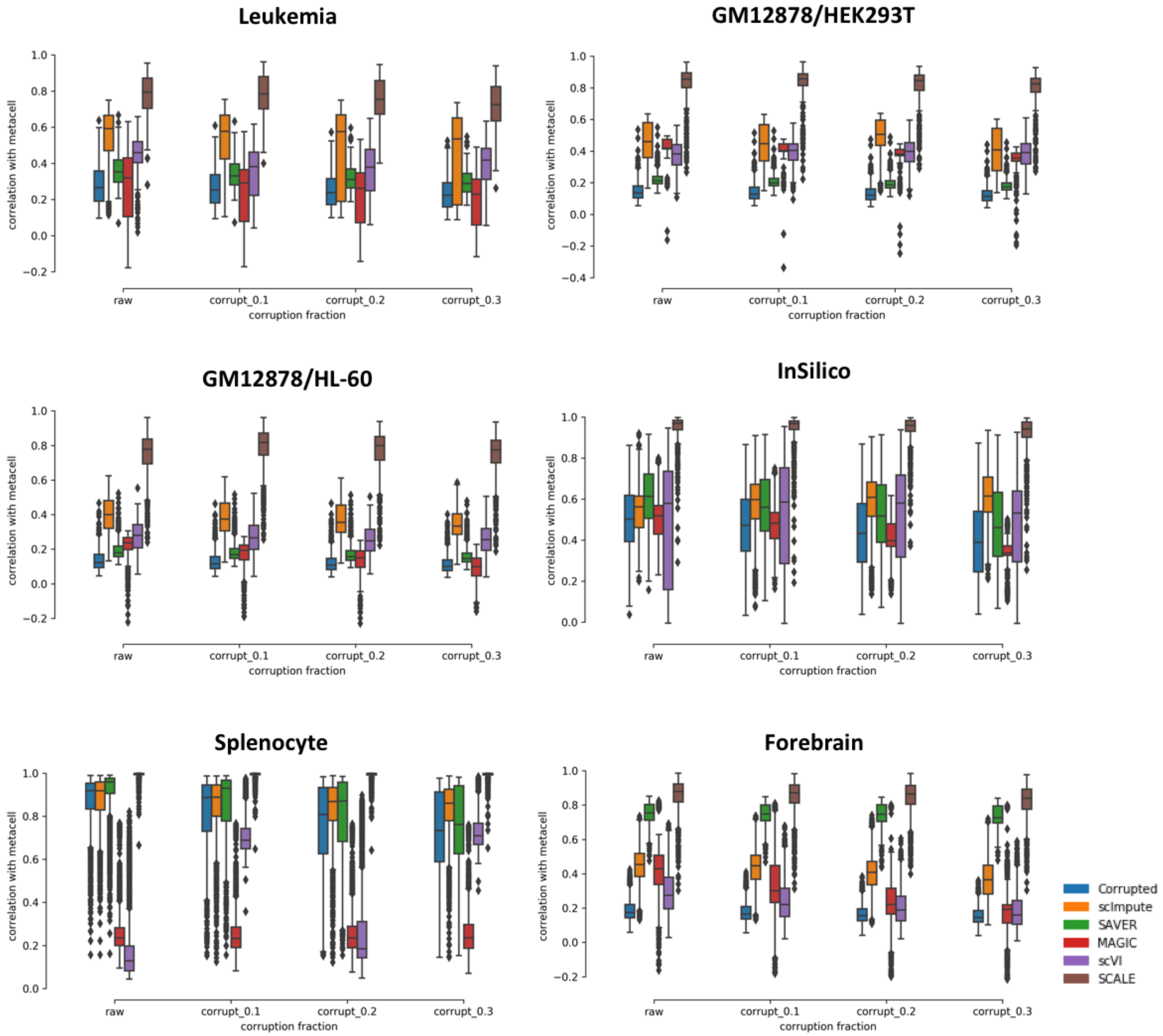

Supplementary Figure 12 | Imputation results on the six real datasets at different corruption levels. Correlation of the imputed cells with the reference meta cells of different cell types for comparison.

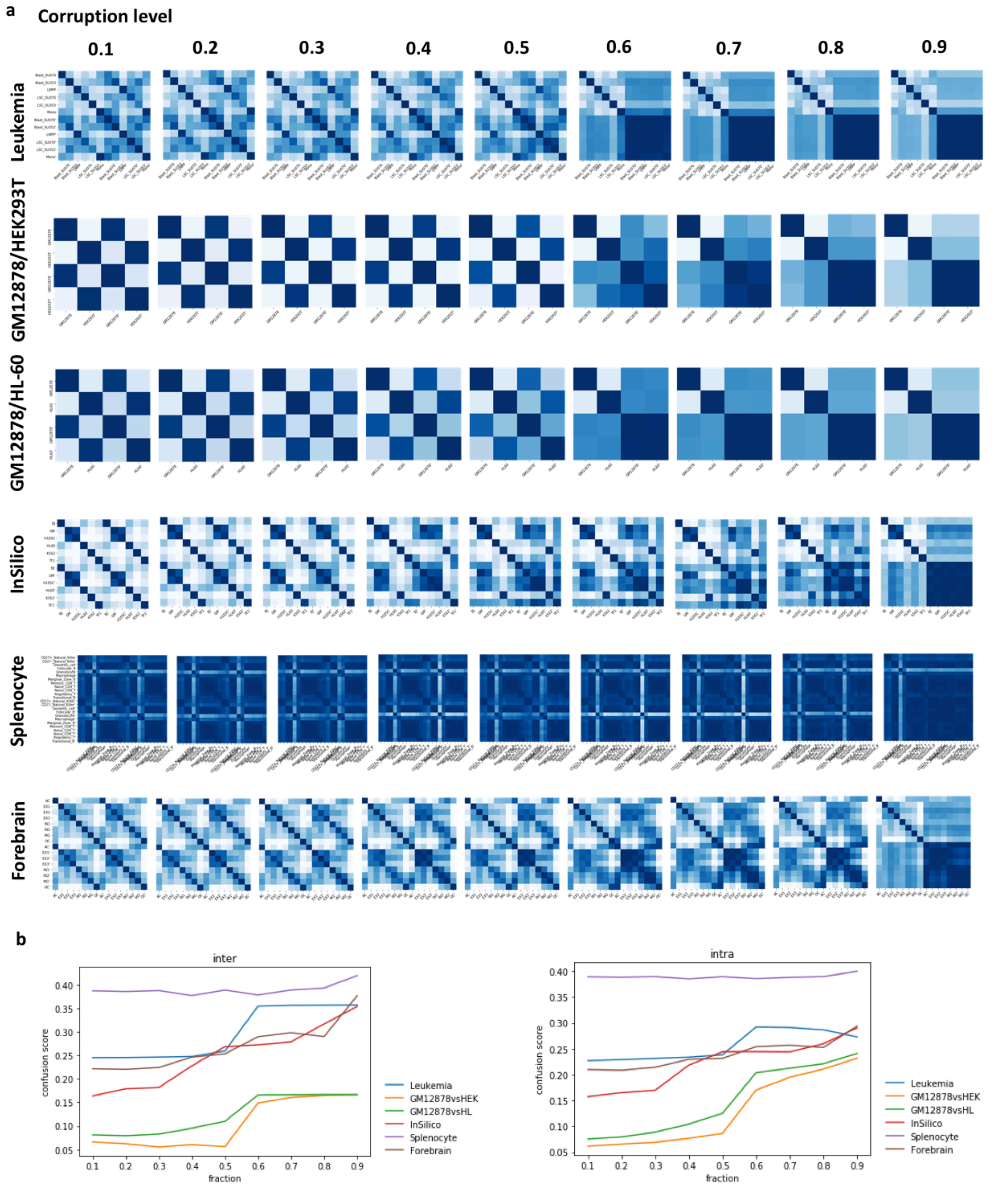

Supplementary Figure 13 | Data corruption impact on preserving original data structure. a, Inter and intra correlation of subgroups of raw and the imputed data at different corruption levels (0.1-0.9). **b,** confusion score of inter and intra correlation.

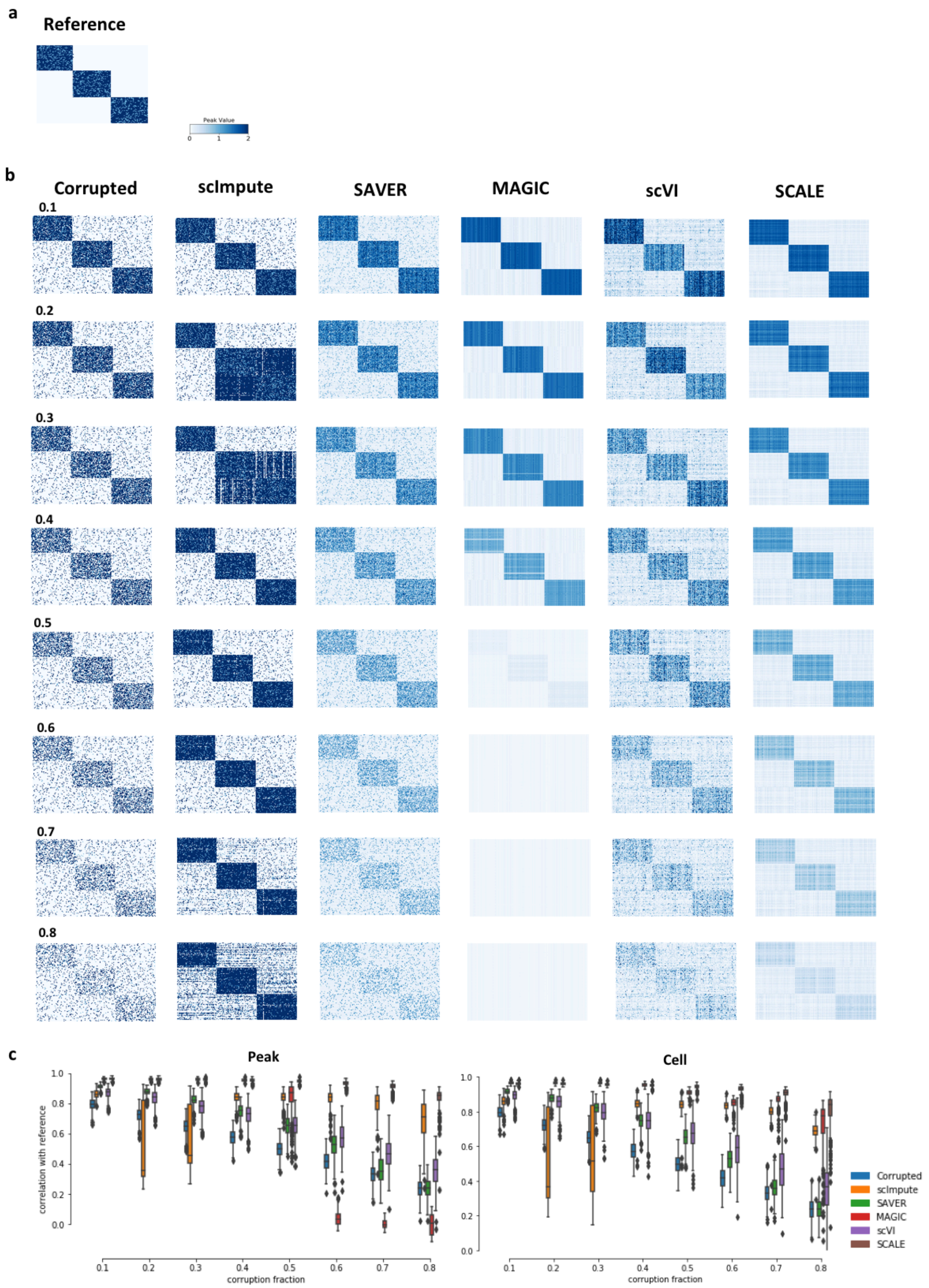

Supplementary Figure 14 | Imputation results on the simulation dataset at different corruption levels. a, Specific peak profile of the simulated reference data. **b**, The corrupted data and the imputed data by scImpute, SAVER, MAGIC, scVI and SCALE at different corruption levels. **c,** Correlation of peak-wise and cell-wise between the imputed cells and the reference.

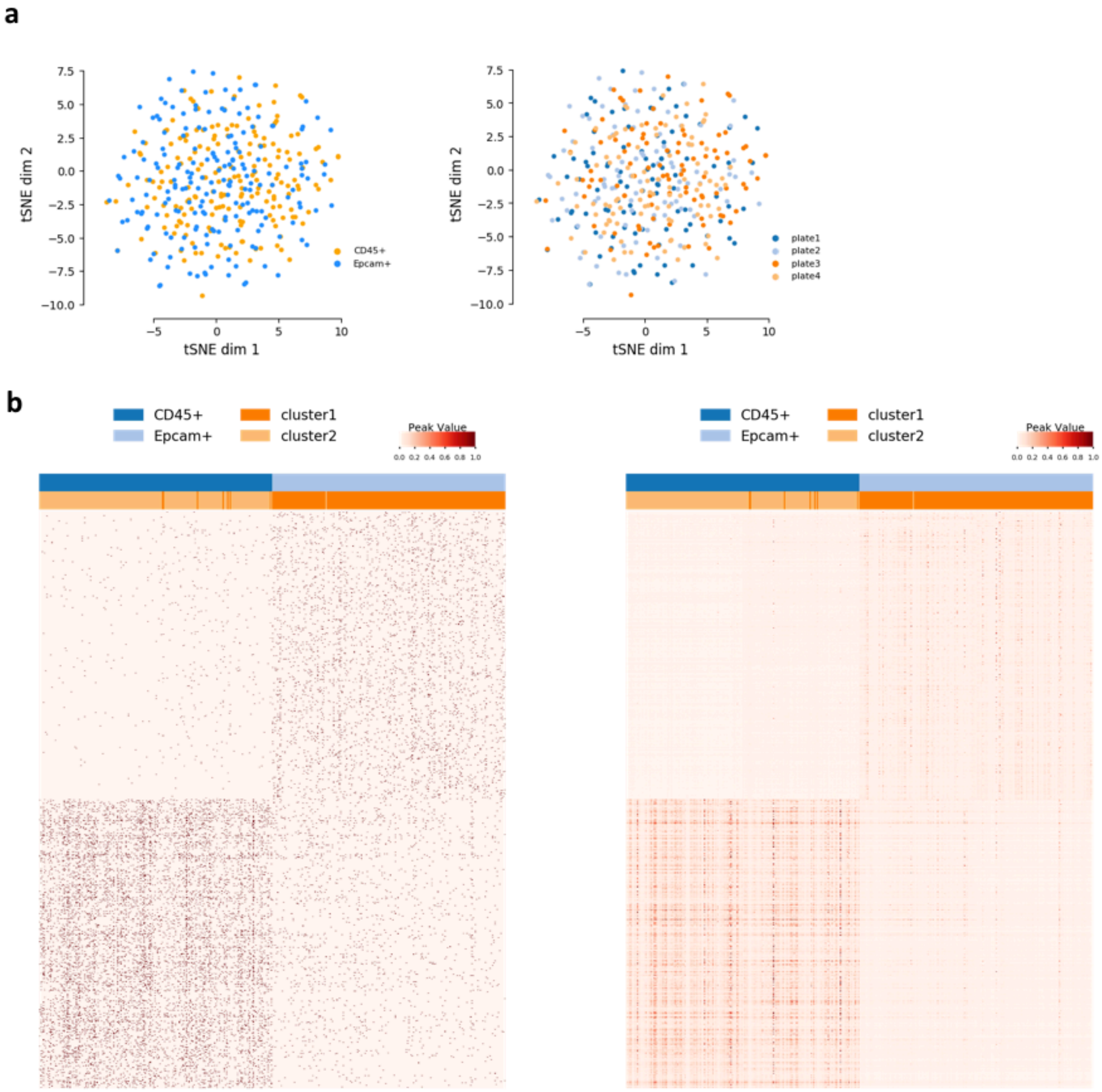

Supplementary Figure 15 | Embedding and specific peaks of the CD45+ and the Epcam+ cells. a, Embedding of the Breast Tumor dataset by the chromVAR analysis method, colored by cell types and plates respectively. Results are similar to those in the original paper (Chen et. al. 2018). **b**, Top 1000 cluster-specific peak profiles from the raw and the imputed data. Ground truth cell labels and predicted cluster assignments are colored on the top**.**

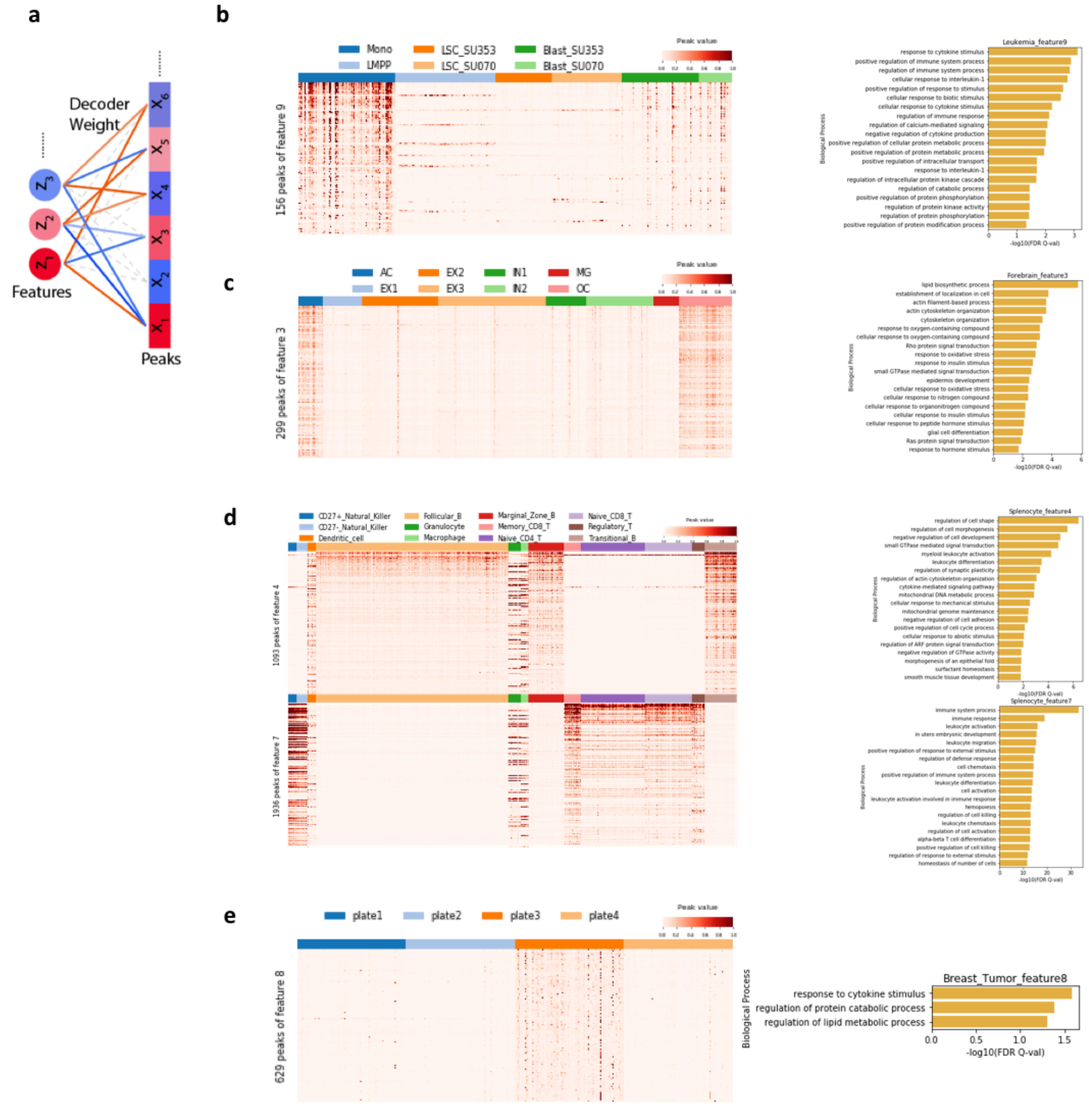

Supplementary Figure 16 | Feature-associated peaks. a, In SCALE, with no latent layer in the decoder network, output peaks are directly linked to the GMM features. **b, c, d, f,** features associated peaks of imputed data and corresponding gene enrichment ('biological process'), **b**, Feature 9 associated peaks were enriched with Mono. **c,** Feature 3 associated peaks were enriched with AC and OC cells. **d,** Features 4 and 7 of associated peaks defined two complementary sets of cell types. **e**, Feature 8 associated peaks were enriched in plate 3.

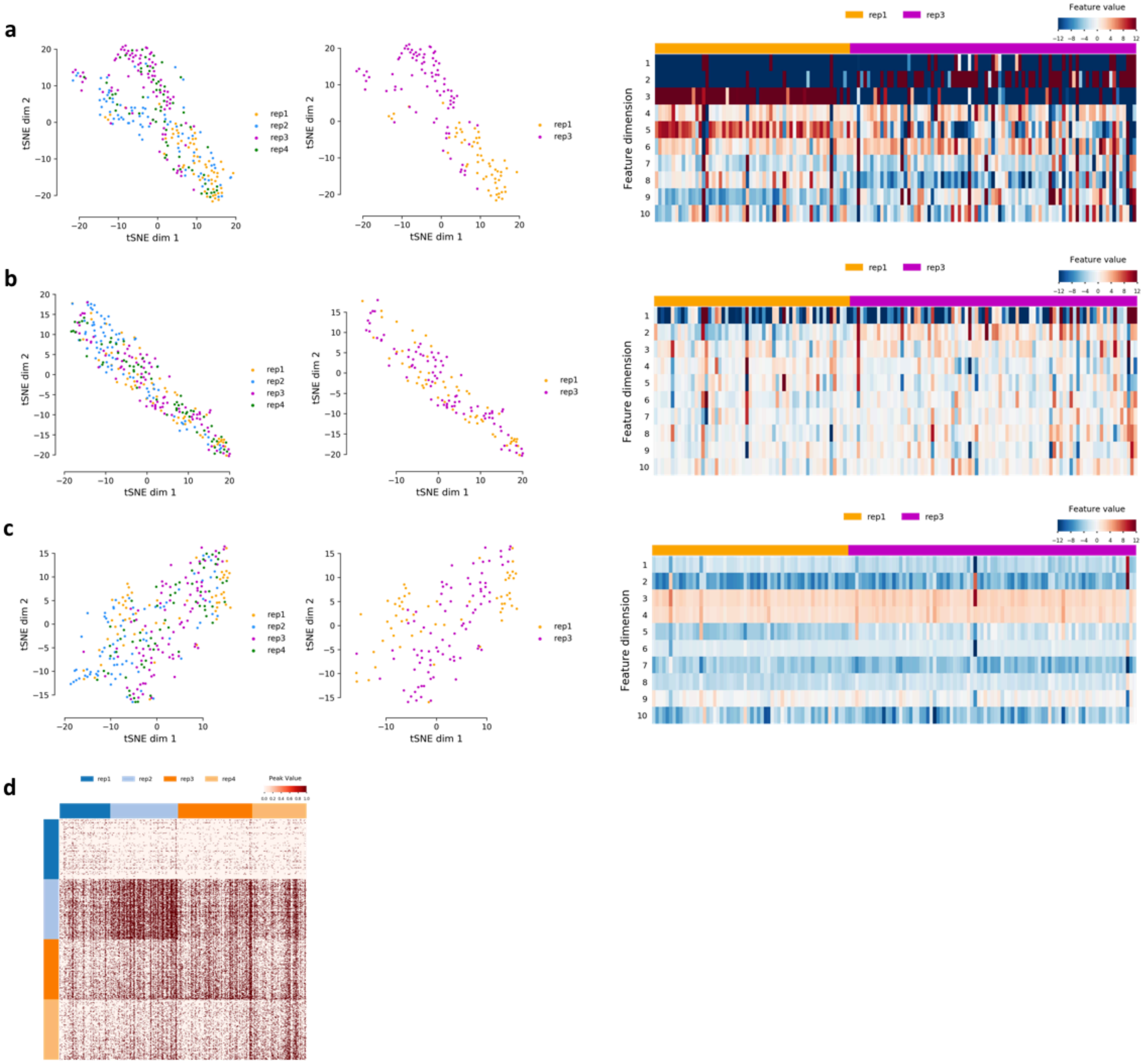

Supplementary Figure 17 | PCA/SCALE extracted features of the GM12878 cells from the InSilico dataset. a, *t*-SNE visualization of the features obtained by PCA colored by replicates and feature heatmap of rep1 and rep3. **b,** *t*-SNE visualization of the feature extracted by PCA from binarized raw data colored by replicates and feature heatmap of rep1 and rep3. **c,** *t*-SNE visualization of the feature extracted by SCALE colored by replicates and feature heatmap of rep1 and rep3. **d,** Heatmap of top 200 specific peaks of each replicates of raw data with colored max values as 1.

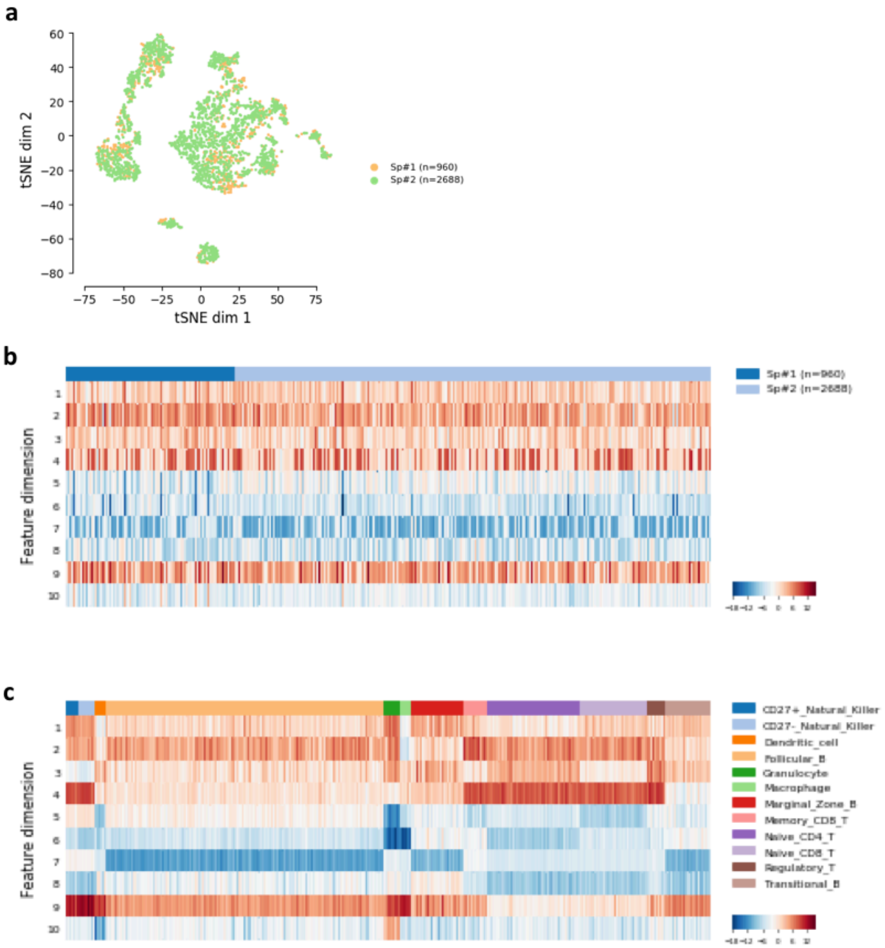

Supplementary Figure 18 | SCALE extracted features of the Splenocyte dataset. a, Feature embedding colored by two replicates. **b,** Feature heatmap with cells grouped by replicates. **c,** Feature heatmap with cells grouped by cell types. Colors represent the association of cell types.

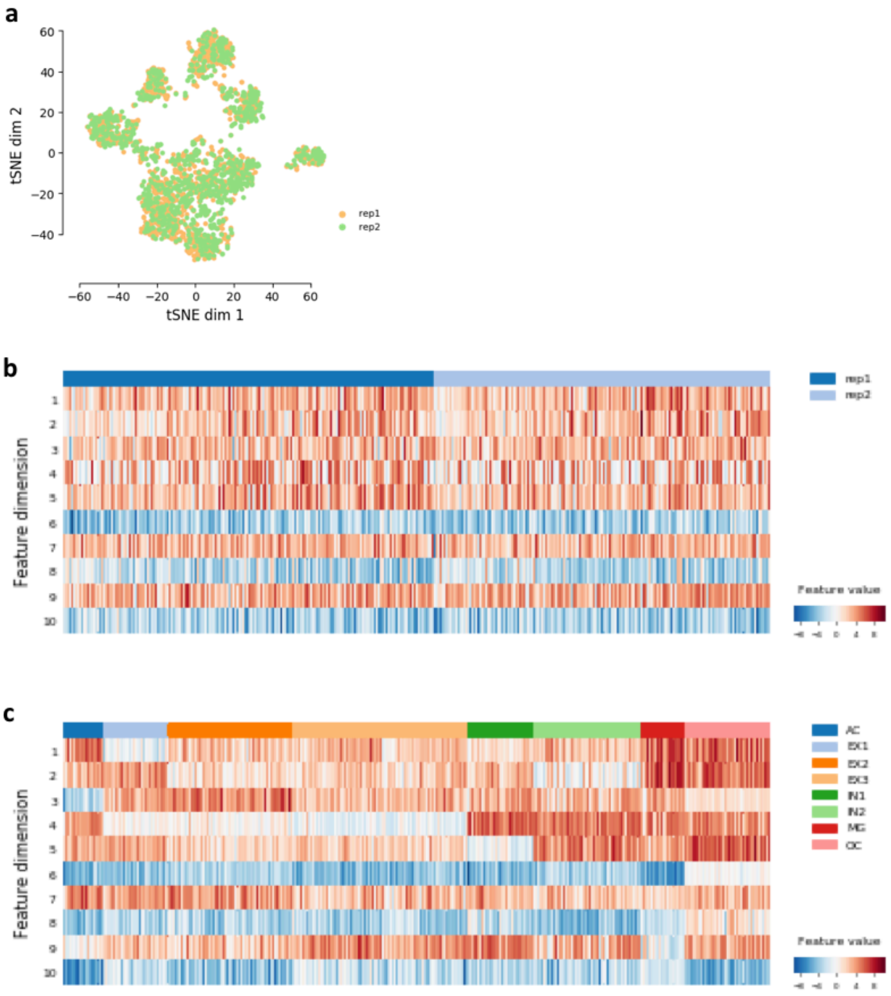

Supplementary Figure 19 | SCALE extracted features of the Forebrain dataset. a, Feature embedding colored by two replicates. **b,** Feature heatmap with cells grouped by replicates. **c,** Feature heatmap with cells grouped by cell types. Colors represent the association of cell types.

a

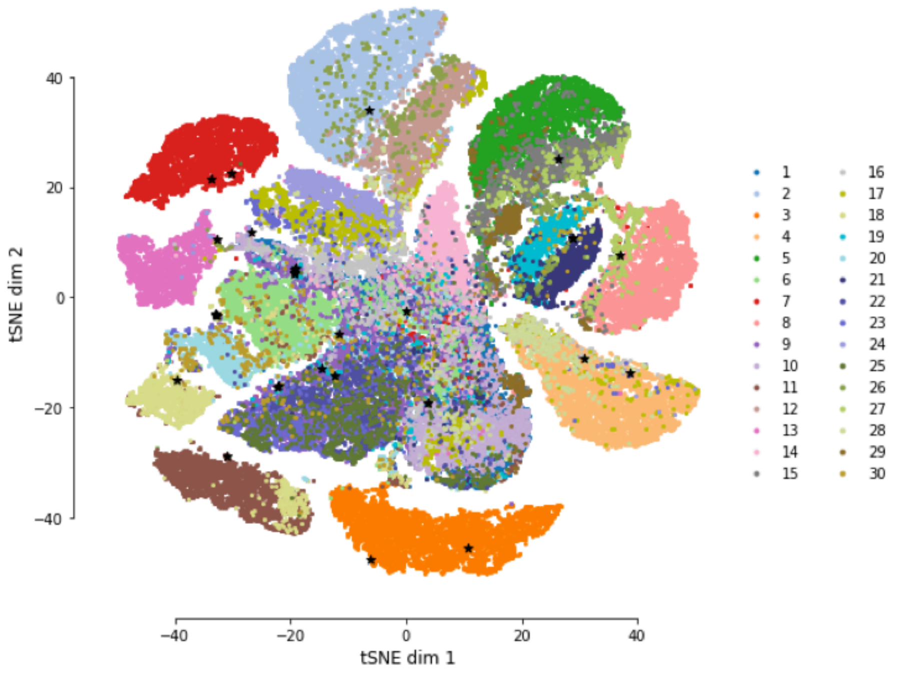

b

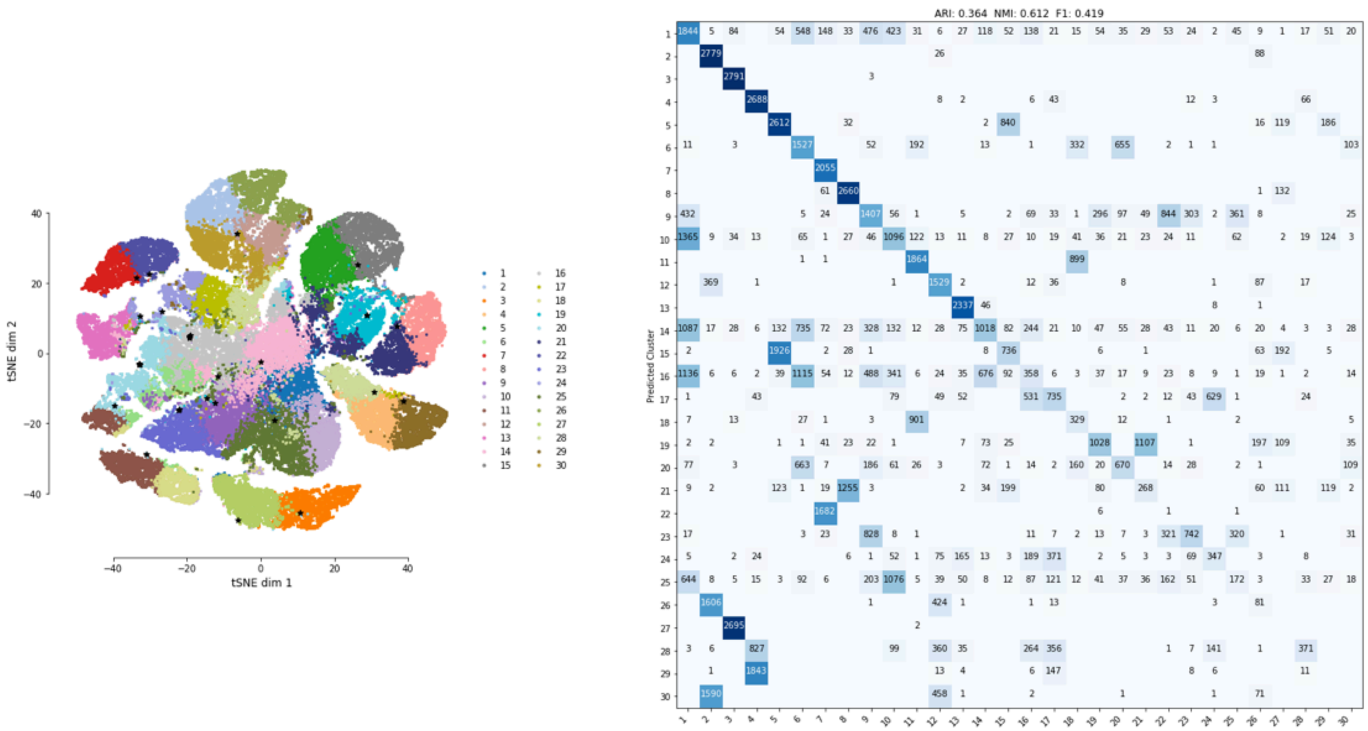

Supplementary Figure 20 | Results of SCALE on a mouse atlas dataset. a, *t*-SNE of SCALE features colored by 30 major clusters in original paper. **b,** cluster assignments by SCALE and confusion matrix with reference 30 major clusters.

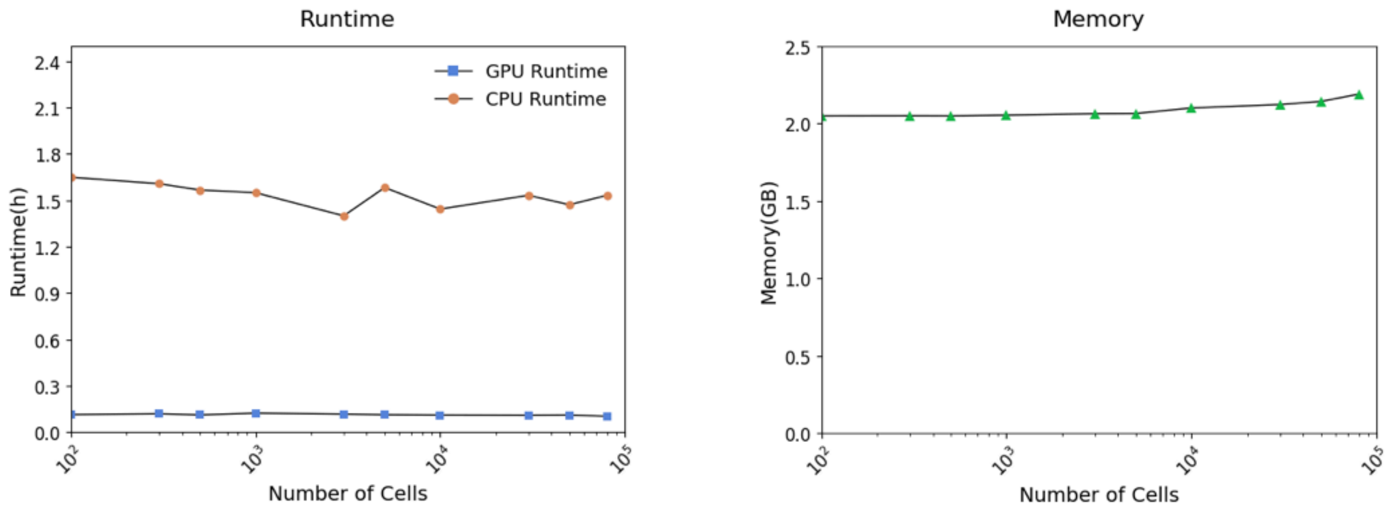

Supplementary Figure 21 | Running time and memory. Running time on dataset down-sampled from mouse atlas datasets (10,000 peaks and different cell numbers) on GeForce GTX 1080 GPU and 1 core CPU and memory usage.

Supplementary Table 1: Results of SCALE with different encoder structures and latent dimensions

	Forebrain			GM12878vsHEK			GM12878vsHL			InSilico			Leukemia			Splenocyte		
Encode_dim&Latent_dim	ARI	NMI	F1	ARI	NMI	F1	ARI	NMI	F1	ARI	NMI	F1	ARI	NMI	F1	ARI	NMI	F1
E128_L8	0.617	0.687	0.823	0.933	0.893	0.964	0.854	0.814	0.935	0.727	0.82	0.754	0.242	0.389	0.481	0.888	0.84	0.971
E128_L10	0.657	0.713	0.845	0.931	0.886	0.964	0.864	0.819	0.936	0.941	0.91	0.96	0.3	0.424	0.558	0.908	0.863	0.977
E128_L12	0.653	0.717	0.842	0.932	0.89	0.964	0.871	0.833	0.938	0.96	0.938	0.976	0.258	0.401	0.468	0.859	0.796	0.964
E128_L15	0.645	0.72	0.839	0.933	0.893	0.964	0.847	0.806	0.933	0.698	0.772	0.744	0.262	0.402	0.491	0.27	0.342	0.76
E128_L20	0.651	0.718	0.841	0.931	0.888	0.964	0.861	0.825	0.936	0.88	0.859	0.923	0.238	0.401	0.476	0.849	0.772	0.961
E128_L50	0.676	0.733	0.854	0.931	0.888	0.964	0.862	0.823	0.936	0.888	0.864	0.926	0.298	0.429	0.54	0.948	0.913	0.987
E1024-128_L8	0.628	0.703	0.83	0.931	0.888	0.964	0.852	0.793	0.933	0.994	0.992	0.998	0.303	0.462	0.496	0.774	0.729	0.94
E1024-128_L10	0.612	0.688	0.818	0.931	0.888	0.964	0.863	0.821	0.936	0.988	0.985	0.995	0.261	0.414	0.471	0.259	0.311	0.755
E1024-128_L12	0.634	0.698	0.83	0.933	0.893	0.964	0.862	0.823	0.936	0.943	0.921	0.96	0.262	0.402	0.491	0.275	0.346	0.763
E1024-128_L15	0.613	0.691	0.82	0.932	0.89	0.964	0.863	0.821	0.936	0.992	0.984	0.995	0.279	0.41	0.514	0.275	0.333	0.763
E1024-128_L20	0.636	0.706	0.832	0.933	0.893	0.964	0.852	0.795	0.933	0.944	0.923	0.961	0.225	0.374	0.478	0.264	0.338	0.758
E1024-128_L50	0.64	0.713	0.838	0.931	0.888	0.964	0.855	0.814	0.935	0.721	0.783	0.795	0.337	0.471	0.563	0.811	0.763	0.951
E1024-256-128_L8	0.613	0.69	0.818	0.931	0.888	0.964	0.842	0.793	0.931	0.997	0.996	0.999	0.221	0.381	0.455	0.83	0.767	0.956
E1024-256-128_L10	0.624	0.691	0.824	0.932	0.89	0.964	0.857	0.809	0.935	0.991	0.989	0.996	0.195	0.376	0.414	0.908	0.851	0.977
E1024-256-128_L12	0.635	0.698	0.831	0.931	0.888	0.964	0.858	0.807	0.935	0.991	0.987	0.996	0.222	0.378	0.465	0.292	0.358	0.771
E1024-256-128_L15	0.629	0.696	0.829	0.931	0.888	0.964	0.849	0.801	0.933	0.987	0.98	0.994	0.337	0.473	0.555	0.259	0.322	0.755
E1024-256-128_L20	0.613	0.686	0.822	0.933	0.893	0.964	0.87	0.835	0.938	0.977	0.967	0.988	0.287	0.428	0.491	0.254	0.318	0.753
E1024-256-128_L50	0.595	0.686	0.809	0.923	0.87	0.962	0.856	0.811	0.935	0.937	0.914	0.955	0.258	0.431	0.44	0.248	0.314	0.75
E1024-512-256-128_L8	0.418	0.54	0.601	0.934	0.897	0.964	0.206	0.289	0.702	0.991	0.987	0.996	0.234	0.398	0.499	0.811	0.728	0.951
E1024-512-256-128_L10	0.505	0.611	0.694	0.931	0.888	0.964	0.855	0.811	0.935	0.983	0.97	0.99	0.327	0.478	0.545	0.765	0.706	0.938
E1024-512-256-128_L12	0.492	0.6	0.713	0.933	0.893	0.964	0.868	0.815	0.936	0.994	0.991	0.998	0.281	0.426	0.522	0.275	0.333	0.763
E1024-512-256-128_L15	0.586	0.664	0.802	0.933	0.893	0.964	0.864	0.821	0.936	0.983	0.971	0.99	0.261	0.393	0.463	0.898	0.851	0.974
E1024-512-256-128_L20	0.541	0.654	0.777	0.932	0.89	0.964	0.846	0.809	0.933	0.994	0.992	0.998	0.253	0.402	0.476	0.248	0.286	0.75
E1024-512-256-128_L50	0.641	0.707	0.837	0.931	0.888	0.964	0.147	0.241	0.67	0.725	0.792	0.795	0.25	0.441	0.419	0.888	0.828	0.971
E3200-400_L8	0.595	0.676	0.806	0.933	0.893	0.964	0.861	0.825	0.936	0.981	0.97	0.99	0.28	0.413	0.504	0.309	0.388	0.779
E3200-400_L10	0.636	0.714	0.834	0.932	0.89	0.964	0.847	0.806	0.933	0.991	0.988	0.996	0.282	0.422	0.509	0.849	0.786	0.961
E3200-400_L12	0.64	0.716	0.836	0.933	0.893	0.964	0.856	0.811	0.935	0.946	0.927	0.964	0.315	0.467	0.527	0.248	0.314	0.75
E3200-400_L15	0.59	0.678	0.809	0.932	0.89	0.964	0.869	0.837	0.938	0.983	0.973	0.99	0.325	0.471	0.527	0.802	0.754	0.948
E3200-400_L20	0.643	0.709	0.836	0.933	0.893	0.964	0.844	0.785	0.931	0.978	0.962	0.984	0.228	0.405	0.412	0.859	0.796	0.964
E3200-400_L50	0.65	0.719	0.841	0.934	0.897	0.964	0.857	0.806	0.935	0.957	0.932	0.965	0.336	0.467	0.514	0.254	0.318	0.753
E3200-800-400_L8	0.617	0.701	0.822	0.933	0.893	0.964	0.806	0.759	0.923	0.991	0.987	0.996	0.208	0.415	0.407	0.711	0.65	0.922
E3200-800-400_L10	0.603	0.677	0.813	0.925	0.874	0.962	0.856	0.808	0.935	0.984	0.976	0.993	0.212	0.358	0.414	0.747	0.68	0.932
E3200-800-400_L12	0.483	0.628	0.682	0.931	0.888	0.964	0.87	0.835	0.938	0.979	0.964	0.988	0.258	0.409	0.483	0.249	0.327	0.75
E3200-800-400_L15	0.547	0.657	0.725	0.932	0.89	0.964	0.856	0.811	0.935	0.994	0.991	0.998	0.27	0.408	0.494	0.275	0.346	0.763
E3200-800-400_L20	0.518	0.616	0.744	0.93	0.885	0.964	0.84	0.799	0.931	0.979	0.968	0.988	0.233	0.412	0.396	0.254	0.318	0.753
E3200-800-400_L50	0.524	0.633	0.731	0.924	0.871	0.962	0.855	0.811	0.935	0.993	0.991	0.998	0.186	0.381	0.384	0.928	0.876	0.982
E3200-1600-800-400_L8	0.502	0.616	0.733	0.932	0.89	0.964	0.268	0.304	0.745	0.966	0.948	0.979	0.207	0.367	0.476	0.508	0.507	0.857
E3200-1600-800-400_L10	0.585	0.666	0.801	0.925	0.874	0.962	0.165	0.263	0.678	0.784	0.871	0.791	0.324	0.472	0.532	0.811	0.749	0.951
E3200-1600-800-400_L12	0.529	0.658	0.764	0.933	0.893	0.964	0.214	0.297	0.707	0.981	0.973	0.992	0.209	0.392	0.417	0.898	0.833	0.974
E3200-1600-800-400_L15	0.475	0.583	0.688	0.934	0.897	0.964	0.841	0.796	0.931	0.988	0.984	0.995	0.312	0.461	0.509	0.783	0.713	0.943
E3200-1600-800-400_L20	0.428	0.585	0.638	0.925	0.877	0.962	0.842	0.793	0.931	0.993	0.987	0.996	0.3	0.472	0.442	0.259	0.311	0.755
E3200-1600-800-400_L50	0.646	0.714	0.837	0.933	0.893	0.964	-0.01	0.004	0.496	0.99	0.983	0.995	0.258	0.448	0.417	0.928	0.876	0.982

Supplementary Table 2: Statistics of scATAC-seq datasets

	n_cells	n_peaks	ref clusters	n_peaks(count=1)	n_peaks(count>=2)	sparsity	platform
Leukemia	391	7602	6	17.3	361.5	0.9502	microfluidics (Fluidigm C1)
GM12878/HEK293T	526	12938	2	46.0	512.6	0.9568	cellular indexing
GM12878/HL-60	597	10431	2	16.0	347.4	0.9652	cellular indexing
In Silico	828	13668	6	85.2	2306.2	0.8250	microfluidics (Fluidigm C1)
Splenocyte	3166	77453	12	1601.5	11266.3	0.8339	microfluidics (FACS)
Forebrain	2088	11285	8	455.4	0.0	0.9596	cellular indexing
Breast_Tumor	384	27884	2	691.5	57.4	0.9731	microfluidics (FACS)
